# Integrative transcriptomic profiling of a mouse model of hypertension-accelerated diabetic kidney disease

**DOI:** 10.1242/dmm.049086

**Published:** 2021-10-25

**Authors:** Frederikke E. Sembach, Helene M. Ægidius, Lisbeth N. Fink, Thomas Secher, Annemarie Aarup, Jacob Jelsing, Niels Vrang, Bo Feldt-Rasmussen, Kristoffer T. G. Rigbolt, Jens C. Nielsen, Mette V. Østergaard

**Affiliations:** 1 Gubra ApS, Hørsholm Kongevej 11B, 2970 Hørsholm, Denmark; 2 Department of Clinical Medicine, University of Copenhagen, Blegdamsvej 3B, 2200 Copenhagen, Denmark; 3 Department of Nephrology, Rigshospitalet, University of Copenhagen, Blegdamsvej 9, 2100 Copenhagen, Denmark

**Keywords:** Diabetic kidney disease, Mouse model, Laser-capture microdissection, Single-nucleus, Glomerulus, RNAseq

## Abstract

The current understanding of molecular mechanisms driving diabetic kidney disease (DKD) is limited, partly due to the complex structure of the kidney. To identify genes and signalling pathways involved in the progression of DKD, we compared kidney cortical versus glomerular transcriptome profiles in uninephrectomized (UNx) *db/db* mouse models of early-stage (UNx only) and advanced [UNxplus adeno-associated virus-mediated renin-1 overexpression (UNx-Renin)] DKD using RNAseq. Compared to normoglycemic *db/m* mice, *db/db* UNx and *db/db* UNx-Renin mice showed marked changes in their kidney cortical and glomerular gene expression profiles. UNx-Renin mice displayed more marked perturbations in gene components associated with the activation of the immune system and enhanced extracellular matrix remodelling, supporting histological hallmarks of progressive DKD in this model. Single-nucleus RNAseq enabled the linking of transcriptome profiles to specific kidney cell types. In conclusion, integration of RNAseq at the cortical, glomerular and single-nucleus level provides an enhanced resolution of molecular signalling pathways associated with disease progression in preclinical models of DKD, and may thus be advantageous for identifying novel therapeutic targets in DKD.

## Abstract (precis)

**Summary:** Combining single-nucleus RNAseq with glomerular and cortical gene expression profiling can link kidney transcriptome signatures to specific cell types in an advanced mouse model of hypertension-accelerated diabetic kidney disease.

## INTRODUCTION

Diabetic kidney disease (DKD) is a microvascular complication of diabetes and the most common cause of chronic kidney disease worldwide, accounting for ∼50% and ∼25% of patients with kidney failure in the USA and EU, respectively (United States Renal Data System, 2018; Kramer et al., 2019). DKD is characterized by the progressive loss of kidney function, as defined by a decline in glomerular filtration rate and proteinuria due to impairment of the glomerular filtration barrier. In addition, patients with DKD demonstrate kidney histopathological alterations, including glomerular hypertrophy, glomerulosclerosis, tubulointerstitial inflammation and fibrosis (Fioretto and Mauer, 2007; Tervaert et al., 2010).

Despite drug therapeutic advances in hyperglycemia and hypertension management (Lewis et al., 1993; Brenner et al., 2001; Wanner et al., 2016; Mann et al., 2017; Perkovic et al., 2019; Heerspink et al., 2020), the combination of increased diabetes prevalence and life expectancy in diabetes patients has led to an increase in patients with DKD progressing to kidney failure (McCullough et al., 2019). Hence, prevention and treatment of DKD has become a healthcare priority. The unmet clinical need is partly due to a lack of animal models that closely mimic the molecular mechanisms associated with late-stage DKD. Hypertension, a common comorbidity in diabetes and a driving factor of DKD progression, is absent in most rodent models commonly used in preclinical DKD research, including the uninephrectomized (UNx) *db/db* mouse model of early-stage DKD (Levine et al., 2008). Induction of persistent hypertension by adeno-associated virus (AAV) delivery of renin-1 (ReninAAV) has recently been reported to accelerate kidney injury in *db/db* UNx mice (Harlan et al., 2018b). Accordingly, the *db/db* UNx-Renin mouse presents with hallmarks of late-stage DKD, including markedly increased urine albumin-to-creatinine ratio (ACR), advanced glomerulosclerosis and elevated serum creatinine levels.

Because the glomerulus has long been considered the primary site of injury in DKD, understanding transcriptome changes in the glomerulus is key from a drug discovery perspective. These investigations are challenged by the fact that glomeruli constitute only a small proportion of renal cortical cells and bulk RNAseq does not capture glomerulus-specific gene expression changes. Therefore, gene expression analysis applied to glomeruli isolated by laser-capture microdissection (LCM) improves the resolution of glomerular transcriptome changes associated with DKD progression (Woroniecka et al., 2011; Levin et al., 2020). This work flow has been applied in drug discovery projects for other metabolic diseases, such as obesity (Paulsen et al., 2009; Zhang et al., 2019).

Although recent developments in transcriptomics enable analysis at the single-cell or single-nucleus level to identify and characterize cell types, these techniques have a low gene detection per cell and a high cost, resulting in limited sensitivity to identify differentially expressed genes (DEGs) at low expression levels (Conesa et al., 2016; Wang et al., 2019). Therefore, an integrative approach leveraging the cell-level resolution of single-nucleus (sn)RNAseq with the sensitivity of bulk RNAseq has proven to be instrumental for gaining detailed insight into the underlying molecular mechanisms involved in specific cell types (Barwinska et al., 2021; Sokolowski et al., 2021).

The current study investigated kidney pathology and disease-related gene and pathway regulations in kidney cortex and glomeruli in mouse models of early-stage (UNx) and advanced (UNx-Renin) DKD. Using a multi-transcriptomic approach, we integrated bulk transcriptomic profiling of kidney cortex and glomeruli with snRNAseq to improve the resolution of cell-specific transcriptomic changes.

## RESULTS

### Diabetic mouse models of early-stage and advanced DKD

**Fig. 1.**
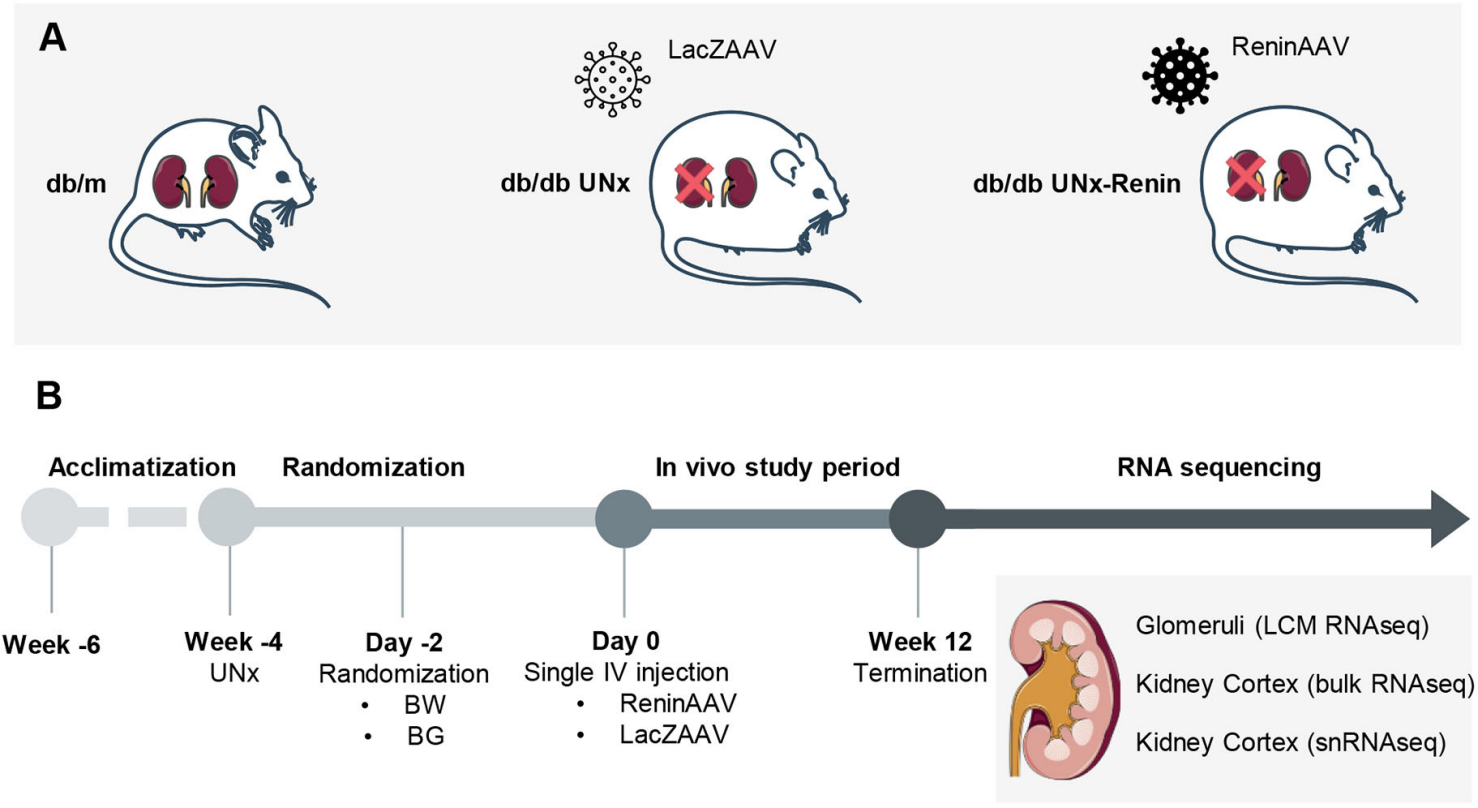
**Schematic of the study outline.** (A) Study groups. (B) Study outline. The top and bottom parts of the kidney cortex were sequestered for bulk RNAseq and snRNAseq. The remaining part of the kidney cortex was cryosectioned and glomeruli were isolated using LCM. BG, blood glucose; BW, body weight.

**Fig. 2.**
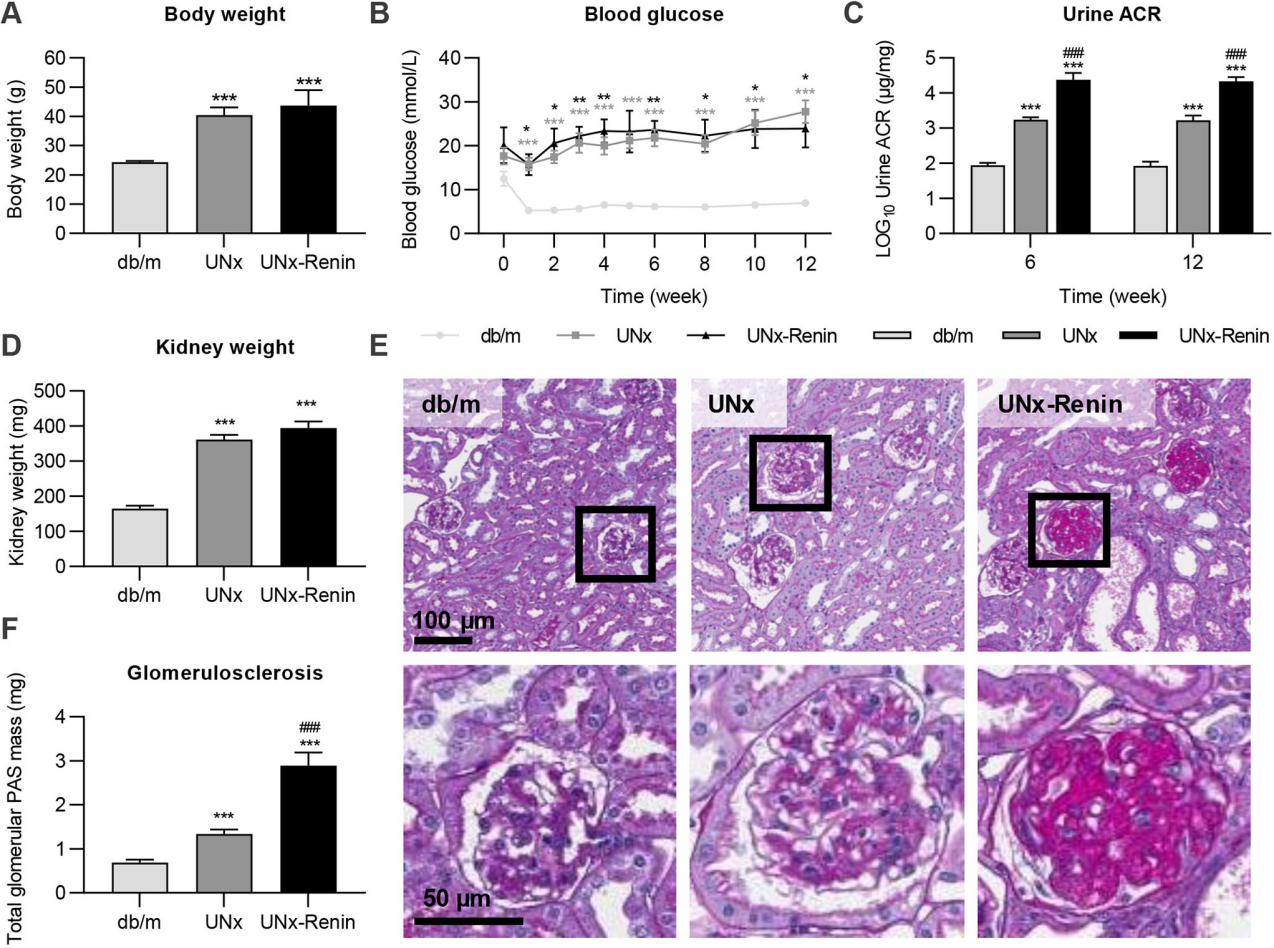
**Measurements 12 weeks after injection with ReninAAV or LacZAAV in diabetic UNx mice and age-matched *db/m* controls.** (A) Body weight. (B) Blood glucose measured biweekly throughout the study. (C) Log10-transformed urine ACR at week 6 and 12 in the study. (D) Kidney weight. (E) Representative images of PAS-stained kidney sections with magnified images of glomeruli below. (F) Quantification of glomerulosclerosis. Data are mean±s.e.m. (*n*=5-13). One-way ANOVA with Tukey's post hoc test (A,D,F) or two-way ANOVA with Bonferroni's post hoc test (B,C). *P<0.05, **P<0.01, ***P<0.001 compared to *db/m*. ^###^*P*<0.001 compared to UNx.

ReninAAV administration was evaluated for renal effects and transcriptome changes in the diabetic UNx mouse model of DKD, whereas UNx mice injected with LacZAAV and healthy *db/m* mice served as controls (Fig. 1A,B). UNx and UNx-Renin mice demonstrated a significantly higher degree of obesity and hyperglycaemia compared to lean *db/m* controls (both *P*<0.001; Fig. 2A,B). Although UNx and UNx-Renin mice showed similar levels of systolic arterial blood pressure at study week 4 (UNx, 137.5±0.9 mmHG; UNx-Renin, 140.9±1.0 mmHg; data are mean±s.e.m.), ReninAAV induced hypertension in UNx *db/db* mice from study week 4 to 10 (+18.8±0.3 mmHg; data are mean±s.e.m.). Urine ACR was significantly increased in UNx mice at study weeks 6 and 12 compared to *db/m* controls (both *P*<0.001), and was further augmented in UNx-Renin mice (both time points *P*<0.001; Fig. 2C). Kidney weight was increased in UNx and UNx-Renin mice compared with *db/m* controls (both *P*<0.001; Fig. 2D). Glomerular hypertrophy was evident in both UNx and UNx-Renin mice (Fig. 2E). Quantification of total glomerular periodic acid–Schiff (PAS) mass indicated increased glomerulosclerosis in UNx and UNx-Renin mice compared to *db/m* controls (both *P*<0.001), being most advanced in UNx-Renin mice (*P*<0.001 versus UNx; Fig. 2F). To summarize, measurements indicate that a single injection with ReninAAV exacerbates kidney injury, notably albuminuria and glomerulosclerosis, in UNx-renin mice compared with UNx mice, but does not affect body weight or blood glucose levels.

To study gene expression changes accelerated by UNx and ReninAAV administration in diabetic mice, RNAseq was performed on kidney cortex samples and laser-capture microdissected glomeruli from *db/m*, UNx and UNx-Renin mice. Comparison of gene expression changes between UNx (early-stage DKD) and UNx-Renin (advanced stage DKD) mice are presented in Fig. S1. A total of 3039 genes were differentially expressed between UNx and UNx-Renin mice in kidney cortex, and 770 genes were differentially expressed in glomeruli between the same groups (Fig. S1). Gene set analysis using the Reactome Pathway Database (Fabregat et al., 2018) identified the immune system as the most significantly affected in the kidney cortex of UNx-Renin mice compared to UNx mice, whereas extracellular matrix (ECM) organization was most affected in glomeruli (Fig. S1).

### Highly specific gene expression profiles in glomeruli versus kidney cortex

**Fig. 3.**
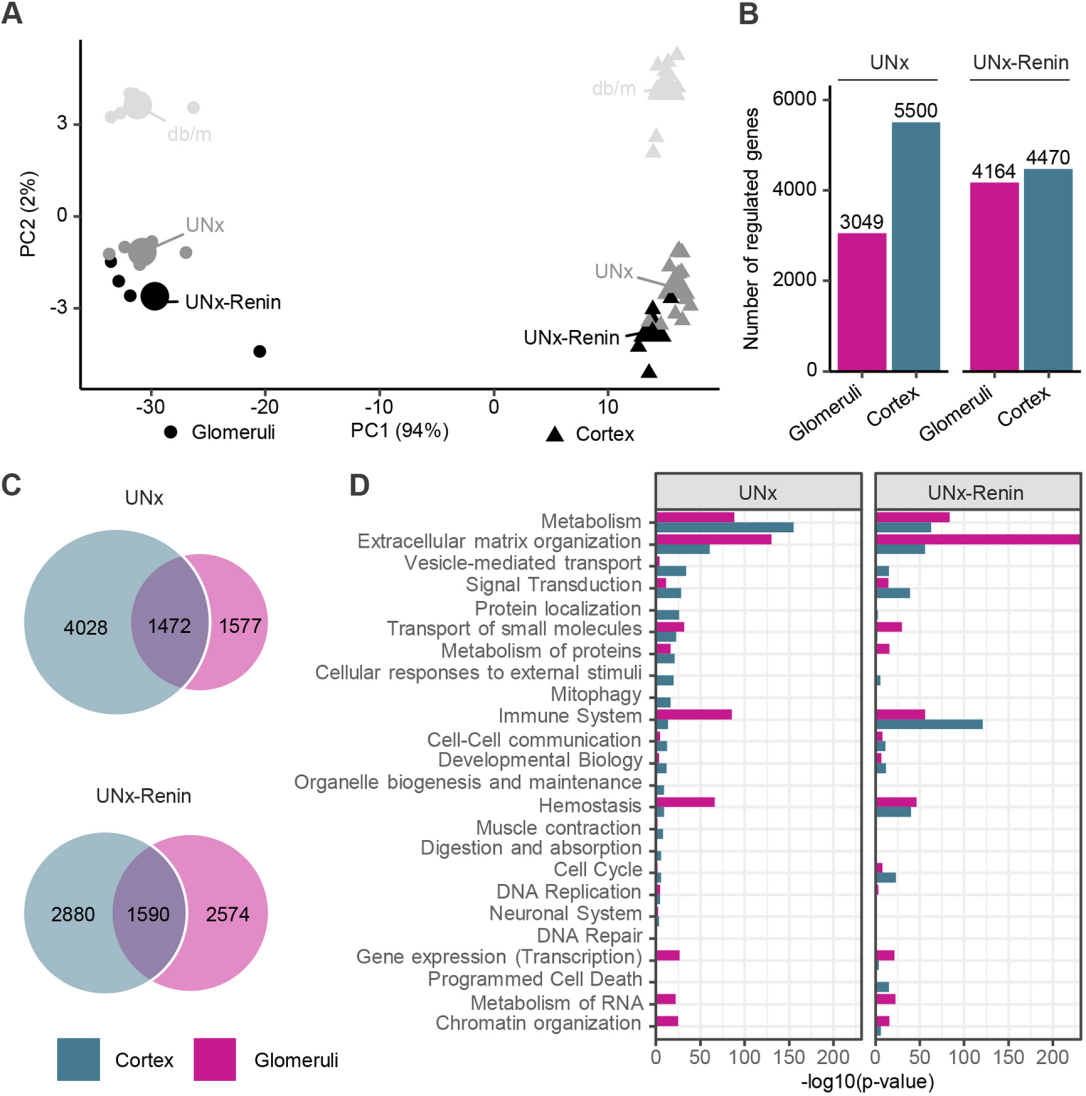
**Highly specific gene expression profiles in glomeruli versus cortex for UNx and UNx-Renin mice compared to *db/m* controls.** (A) PCA of the 500 most variable genes. Small points indicate a sample and large points the group centre. (B) Total number of DEGs in glomeruli and kidney cortex from UNx and UNx-Renin mice compared with *db/m* controls. (C) Venn diagrams depicting shared and separate DEGs in glomeruli and kidney cortex from UNx or UNx-Renin mice. (D) Reactome pathway gene enrichment analysis in glomeruli and kidney cortex from UNx or UNx-Renin mice. Degree of perturbation is presented as the −log10(*P*-value) after correction for gene-wise multiple testing (*n*=5-13).

To characterize glomerular-specific gene expression changes in the UNx and UNx-Renin mouse models of DKD, we compared the glomerular transcriptome with the full kidney cortex transcriptome. First, we performed a principal component analysis (PCA), which is a dimensionality reduction method used to assess the similarity of the gene expression profiles from individual samples, with samples closest to each other being most similar. The PCA demonstrated a clear separation between the glomerular transcriptome signature and kidney cortex from *db/m*, UNx and UNx-Renin mice (Fig. 3A). The difference between isolated glomeruli and kidney cortex samples explained the majority of the variance in the data set as indicated by a percentage (94%, PC1), followed by the difference between phenotypes (*db/m*, UNx, UNx-Renin, 2%, PC2; Fig. 3A). Gene expression analysis of UNx and UNx-Renin mice compared to healthy *db/m* controls, revealed a higher number of DEGs in the kidney cortex of UNx mice (5500 DEGs) than in UNx-Renin mice (4470 DEGs; Fig. 3B). The opposite was seen for the glomerular transcriptome (UNx, 3049 DEGs; UNx-Renin, 4164 DEGS; Fig. 3B). Interestingly, few DEGs overlapped between kidney cortex and isolated glomeruli for both UNx (1472 DEGs) and UNx-Renin (1590 DEGs) mice compared to healthy *db/m* controls (Fig. 3C). Overall, these data substantiate that RNAseq of kidney cortex does not specifically reflect glomerular gene expression changes in both the UNx and UNx-Renin models of DKD, and highlights the importance of profiling the transcriptome of kidney structures separately.

A gene set enrichment analysis was performed to assess changes in signalling pathways specific to the UNx and UNx-Renin groups, including both upregulated and downregulated genes in the signalling pathways (Fig. 3D; Fig. S2). Compared to *db/m* controls, metabolism was the most regulated pathway in the kidney cortex of UNx mice, predominantly driven by increased expression of *Rbp2*, *Cyp2d9* and *Ugt1a10* (Table S1). In contrast, changes in cortical gene signatures of UNx-Renin mice were associated with the immune system, including both the innate immune system (neutrophil degranulation and toll-like receptor cascades) and cytokine signalling (signalling by interleukins and the TNFR2 non-canonical NF-kB pathway; Fig. S2). Concordantly, increased expression of chemokines (*Cxcl1* and *Cxcl2*), vascular cell adhesion molecule (*Vcam1*), toll-like receptor (*Tlr4*) and complement component genes (*C3*, *C6* and *C7*) were observed in UNx-Renin mice. Glomerular gene expression signatures in UNx and UNx-Renin mice were mainly associated with ECM organization (Fig. 3D), as exemplified by the increased expression of several genes involved in fibrogenesis (e.g. *Col5a1*, *Col5a3*, *Fn1*, *Lox*, *Mmp3*, *Mmp8*, *Mmp12*, *Adamts4*, *Serpine 1* and *Timp1*; Table S2). Consistent with marked glomerulosclerosis in UNx-Renin mice, ECM-associated gene expression changes were more pronounced in UNx-Renin mice, in which additional markers of fibrogenesis were significantly upregulated, including *Col1a1*, *Col3a1*, *Col6a1*, *Col6a2* and *Mmp7*.

### snRNAseq reveals glomerular cell-specific regulations in DKD pathogenesis

**Fig. 4.**
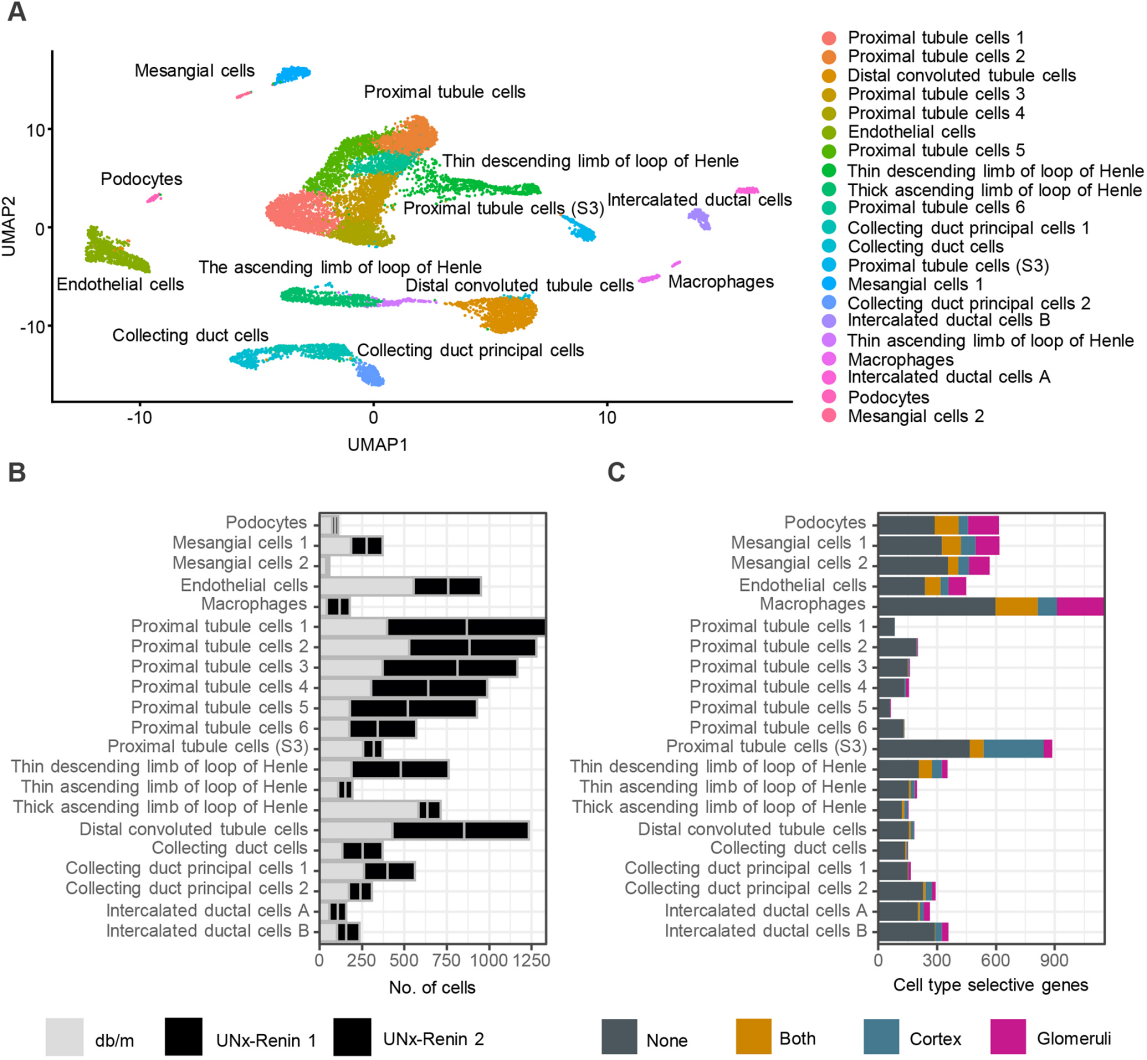
**Number of DEGs in cortex and glomeruli of UNx-Renin mice mapped to a specific cell type using snRNAseq.** (A) UMAP of 12,840 nuclei from mainly kidney cortex of *db/m* and UNx-Renin mice (*n*=1-2). Each dot represents a nucleus coming from a single cell. Cells that show similar transcriptomic profiles are grouped by colour based on unsupervised clustering. A total of 21 cell populations were identified. (B) Number of cells found in each cell population per animal. (C) Number of cell type-specific genes significantly regulated between UNx-Renin and *db/m* mice in glomeruli, cortex, both or none of the two tissue areas. Genes were defined as specific to a cell population if the expression was increased by twofold compared to the cell population with the second highest expression level.

To further investigate the marked transcriptomic changes between normoglycemic *db/m* controls and diabetic hypertensive UNx-Renin mice, we performed snRNAseq to associate gene expression changes with specific cell types harvested from the kidney cortex. Unsupervised clustering identified 21 cell clusters. Based on canonical cell-type markers each cluster was assigned to distinct cell types. Fig. 4A shows the projection of single nuclei onto two-dimensional uniform manifold approximation and projection (UMAP) space coloured by assigned cell types. In addition to all major cortical cell types, such as podocytes, mesangial cells, endothelial cells, macrophages, proximal tubule cells, distal tubule cells and collecting duct cells, we detected specific gene signatures of loop of Henle-associated cell types, indicating the presence of medullary cells in the samples. Our preliminary analysis suggested reduced podocyte and endothelial cell proportions in UNx-Renin mice (Fig. 4B) compared to *db/m* control.

**Fig. 5.**
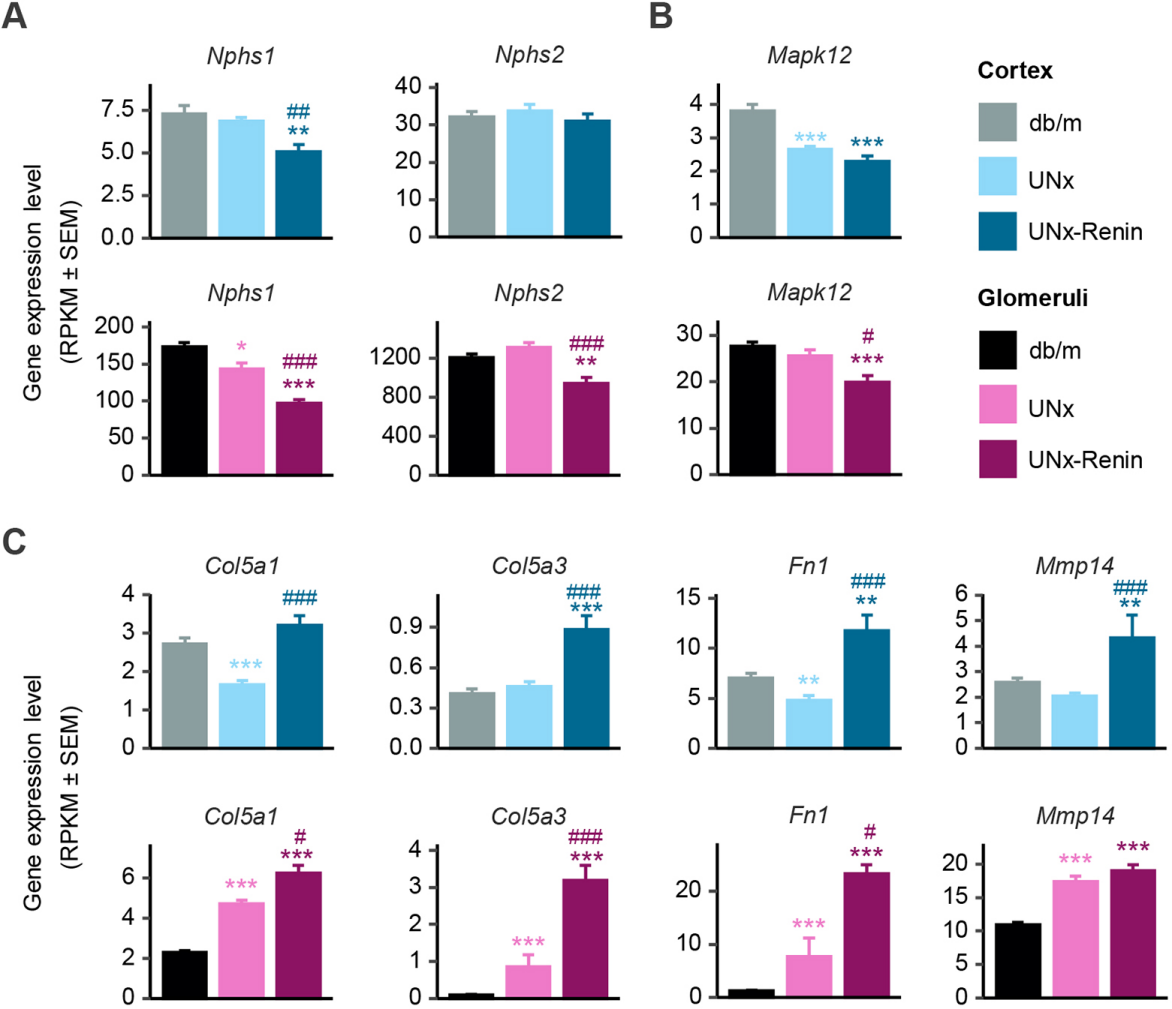
**Regulation of cell type-specific genes in cortex and glomeruli of UNx-Renin mice.** (A-C) Expression levels presented as mean±s.e.m. RPKM values for genes in nephrin family interactions (*Nphs1* and *Nphs2*) (A), myogenesis (*Mapk12*) (B) and degradation of ECM (*Col5a1*, *Col5a3*, *Fn1* and *Mmp14*) (C) pathways. *P<0.05, **P<0.01, ***P<0.001 compared to *db/m*. ^#^*P*<0.05, ^##^*P*<0.01, ^###^*P*<0.001 compared to UNx (false discovery rate adjusted *P*-values, *n*=5-13).

The combination of snRNAseq data with kidney cortex and LCM-isolated glomeruli-based RNAseq data allowed us to associate DEGs to specific cell types in UNx-Renin mice (Fig. 4C). We found that a large proportion of cortical DEGs in UNx-Renin mice were linked to cells in the S3 segment of the proximal tubule. In contrast, glomerular DEGs identified were distributed more broadly, including podocytes, mesangial cells, macrophages and endothelial cells. Furthermore, we superimposed DEGs found between UNx mice and *db/m* controls, or between UNx-Renin and UNx mice, to specific cell types in Fig. S3. Consistent with gene expression changes in the immune system pathway in the kidney cortex between UNx-Renin and UNx mice (Fig. S1), cortical DEGs identified between the two models were linked to macrophages. Finally, a gene set analysis was applied to characterize changes in signalling pathways associated with podocytes, endothelial cells and mesangial cell populations in UNx and UNx-Renin mice (Figs S4-S6). Key genes driving pathway changes are presented in Fig. 5.

In UNx-Renin mice, podocyte markers were downregulated in the kidney cortex (*Nphs1*, *P*<0.01 compared to *db/m* controls) and glomeruli (*Nphs1* and *Nphs2*, *P*<0.001 versus *db/m*; *P*<0.001 and *P*<0.01 versus UNx; Fig. 5A), indicating podocyte loss. Compared to *db/m* controls, both UNx and UNx-Renin mice showed downregulation of the endothelial cell marker *Mapk12* in the kidney cortex (both *P*<0.001 versus *db/m* controls), whereas *Mapk12* expression was only downregulated in glomeruli of UNx-Renin mice (*P*<0.001 versus *db/m*, and *P*<0.05 versus UNx mice; Fig. 5B). However, overall our gene set analysis of endothelial cell markers showed more pronounced changes in UNx mice than in UNx-Renin mice compared to *db/m* controls, suggesting that endothelial cell injury may occur in the early stage of DKD (Fig. S5).

*Col5a1*, *Col5a3*, *Fn1* and *Mmp14* expression was associated with mesangial cells, supporting increased ECM production in glomeruli. *Col5a3*, *Fn1* and *Mmp14* were upregulated in the kidney cortex and glomeruli of UNx-Renin mice compared to both *db/m* controls and UNx mice (Fig. 5C), underscoring the advanced progression of glomerulosclerosis in this model of late-stage DKD.

## DISCUSSION

We report highly different glomerular and cortical transcriptome changes in mouse models of early-stage (UNx) and advanced (UNx-Renin) DKD. The transcriptome signatures identify metabolic and immune responses as essential disease drivers in UNx and UNx-Renin mice, respectively, and glomerular ECM regulation is seen in both models but most prominently in UNx-Renin mice. This is consistent with the advanced glomerulosclerosis in the model. Our study highlights the utility of combining bulk RNAseq with snRNAseq to define cell type-specific gene regulations important for DKD progression and thus further enabling the identification of novel drug targets. Additionally, the gene expression profiling of our two mouse models of DKD will allow researchers to select the most appropriate preclinical model for drug testing.

The diabetic *db/db* UNx mouse model of DKD has been well characterized since the model was first introduced in 1980 (Bower et al., 1980). Although the *db/db* UNx mouse presents with mesangial matrix expansion and increased albuminuria, the model does not capture the decline in glomerular filtration rate, as often seen in patients with advanced DKD (Bower et al., 1980; Ninichuk et al., 2007; Levine et al., 2008; Sembach et al., 2019). The lack of decline in glomerular filtration rate may be caused by the absence of hypertension in the model (Levine et al., 2008), which is a common comorbidity in DKD (Long and Dagogo-JAck, 2011). The UNx-Renin mouse represents a promising model of progressive DKD (Harlan et al., 2018b). Accordingly, ReninAAV administration induces persistent hypertension and has recently been shown to accelerate DKD progression in diabetic UNx mice (Harlan et al., 2018b; Østergaard et al., 2021). Our study corroborates previous findings in the UNx-Renin model, demonstrating biochemical and histological features of progressive DKD, notably severe albuminuria and advanced glomerulosclerosis (Harlan et al., 2018a; 2018b; Østergaard et al., 2021). Although several transcriptomic studies have been reported in the *db/db* UNx and UNx-Renin mouse models, knowledge on disease-associated gene expression changes in these models is based on preselected gene sets (Ninichuk et al., 2007; Harlan et al., 2018a,b). Consequently, global gene expression profiling could help validate the clinical translatability of these models. In this report, we characterize the global kidney transcriptome changes in the UNx and UNx-Renin mouse models using RNAseq.

As expected, RNAseq data revealed a clear separation of cortical versus glomerular transcriptome signatures, reflecting unique transcriptional signatures in glomeruli that were not shared in the kidney cortex, as exemplified by the expression of *Nphs1*, *Nphs2*, *Col5a3* and *Mmp14*. These genes were significantly regulated in the glomeruli of UNx mice compared to healthy *db/m* controls but not in the kidney cortex. Because bulk RNAseq analysis captures a composite gene expression signal from the whole tissue, hence from all cell types in the sample, the low number of glomerular cells compared to other kidney cortical cell types cannot be captured.

In the present study, gene expression changes associated with the immune system were prominent in the kidney cortex in diabetic UNx-Renin mice, whereas diabetic UNx mice demonstrated more robust changes in metabolism-associated genes compared to healthy *db/m* controls. This is consistent with previous studies reporting increased interstitial inflammation along with the upregulation of inflammatory markers (e.g. *C3* and *Cxcl2*) in UNx-Renin mice compared to UNx mice (Harlan et al., 2018a,b). Collectively, these findings are in agreement with pronounced kidney immune system responses in patients with advanced DKD compared to early-stage DKD (Fan et al., 2019). In combination, snRNAseq and bulk RNAseq analysis further emphasized that differences in cortical immune system changes in diabetic UNx-Renin compared to UNx mice were mainly associated with gene markers of macrophage recruitment. Correspondingly, kidney macrophage infiltration has been shown to correlate with impaired kidney function in patients with DKD (Nguyen et al., 2006). We found an increased expression of *Cxcl1* and *Cxcl2*, encoding proinflammatory chemokines that stimulate monocyte infiltration (Pichler et al., 2017), and of *C3*, *C6* and *C7* genes, which are part of the complement system known to be activated in human DKD (Woroniecka et al., 2011; Ju et al., 2013; Sircar et al., 2018; Yiu et al., 2018). Additionally, a negative correlation between C3 upregulation in tubulointerstitium and decreased glomerular filtration rate has previously been shown in patients with DKD (Tang et al., 2020). Upregulation of *Vcam1* in UNx-Renin mice has been reported in other experimental mice models of DKD (KK-Ay, BTBR *ob/ob*) (Omote et al., 2014; Granqvist et al., 2020), and is known to recruit leukocytes to the site of inflammation. In agreement, an upregulation of VCAM-1 in tubulointerstitium, together with a positive correlation between elevated serum VCAM-1 and albuminuria levels, have been reported in DKD patients (Schmid et al., 2006; Wong et al., 2008). Immune responses in UNx-Renin mice were also characterized by the upregulation of markers associated with toll-like receptor (TLR) signalling, as exemplified by the increased gene expression of *Tlr4* in kidney cortex and glomeruli of UNx-Renin mice compared to healthy *db/m* controls. Concordantly, gene and protein expression of TLR4 is upregulated in glomeruli and tubulointerstitium of patients with DKD (Verzola et al., 2014). In support of the relevance of TLRs in DKD, inhibition of TLR signalling has been shown to improve albuminuria in mouse models of DKD (Cha et al., 2013; Lin et al., 2013). Overall, our data suggest that the *db/db* UNx-Renin mouse model recapitulates human DKD in terms of immune response and indicate inflammatory pathway activation as key to the pathogenesis.

The glomerulus has long been considered the primary site of injury during DKD progression due to changes in the glomerular structure, such as mesangial expansion, glomerular basement membrane thickening and glomerulosclerosis (Fioretto and Mauer, 2007; Tervaert et al., 2010; Woroniecka et al., 2011). As molecular drivers of glomerulosclerosis in diabetic UNx and UNx-Renin mice, our glomerular gene expression analysis confirmed the upregulation of several fibrogenesis-associated genes (e.g. *Col1a1*, *Col5a1*, *Col5a3*, *Fn1* and *Mmp14*). These results are consistent with previous studies in microdissected glomeruli from patients diagnosed with diabetic nephropathy that showed increased expression of *Col1a1*, *Col5a1* and *Fn1* (Woroniecka et al., 2011; Levin et al., 2020). Fibronectin (*Fn1*) has been particularly linked to the accumulation of ECM proteins, and treatment with the SGLT2 inhibitor canagliflozin in individuals with type 2 diabetes and elevated urine ACR has been shown to downregulate plasma levels of fibronectin, thereby reducing the fibrosis biomarker (Heerspink et al., 2019). Also, the expression of *Col1a1* is upregulated in human DKD glomeruli (Woroniecka et al., 2011; Ju et al., 2013; Levin et al., 2020). Using snRNAseq analysis, we found that most DEGs involved in fibrogenesis were associated with mesangial cells, confirming that mesangial cells are critical players in DKD progression and may be potential targets for the prevention and treatment of glomerulosclerosis. In UNx-Renin mice, we identified nephrin family interactions as being the top-regulated pathway in podocytes, primarily associated with the downregulation of the key podocyte markers *Nphs1* and *Nphs2*. Downregulation of these genes is consistent with the podocyte loss observed in *db/db* mice (Susztak et al., 2006). *NPHS1* and *NPHS2*, together with several other podocyte-specific genes, are also downregulated in human DKD studies (Woroniecka et al., 2011), emphasizing the role podocyte injury plays in DKD pathogenesis. Our analysis demonstrates higher expression levels of podocyte-specific genes in glomeruli compared to the kidney cortex, highlighting the advantage of isolating glomeruli for gaining further insight into gene expression changes associated with specific glomerular cell types.

In contrast, we found that several endothelial cell-specific genes were regulated in the kidney cortex of UNx mice compared to *db/m* controls, whereas this was not the case with UNx-Renin mice, suggesting that changes in the microvasculature occur during the early stages of DKD. Similarly, features of endothelial dysfunction and changes in angiogenesis have been observed in patients with early DKD (Jensen et al., 1989; Wilson et al., 2019).

Limitations in the study should be considered. First, snRNAseq analysis was limited by the small sample size. Therefore, we integrated the specificity of snRNAseq with the sensitivity of bulk RNAseq to assign gene expression changes to individual kidney cell types. A similar approach has recently been used for bulk RNAseq to quantitively characterize transcriptomic profiles with single-cell resolution (Ægidius et al., 2020; Dong et al., 2021) or to infer cell type proportions by bulk RNAseq deconvolution (Fan et al., 2019). In our study, we captured all major cell types in the kidney except for glomerular endothelial cells, which have been observed in previous studies (Fu et al., 2019; Jourde-Chiche et al., 2019). The lack of glomerular endothelial cell gene expression signatures might be due to bias towards specific cell types during nuclei isolation, or the resolution of the clusters used for the bioinformatical analysis. Therefore, further sub-analysis of the endothelial cell population is needed to confirm whether glomerular endothelial cells are captured. Combining bulk RNAseq with snRNAseq constitutes a powerful tool to detect cell-specific changes. Notably, it provided us with cell type-specific DEGs and specific pathway regulations in glomerular cell types (e.g. podocytes, mesangial cells and endothelial cells).

In conclusion, the present study allowed the detection of gene expression changes specific to glomeruli using LCM of kidney cortical samples. The transcriptomic changes identified in the *db/db* UNx-Renin mouse model support histological hallmarks of progressive DKD in this model. Combining snRNAseq with RNAseq of glomeruli and kidney cortex enables further resolution of the transcriptome signatures with respect to kidney cell types. Using this approach, the integration of different RNAseq methods has the potential to improve future drug discovery activities, including the selection of an appropriate preclinical mouse model of DKD.

## MATERIALS AND METHODS

### Animals

The Danish Animal Experiments Inspectorate approved all experiments, which were conducted using internationally accepted principles for the use of laboratory animals (2018-15-0201-01533). Female C57BLKS *db/db* (BKS.Cg-Dock7*^m^*+/+Lepr*^db^*J) and *db/m* mice (Dock7m+/+Leprdb) (Charles River Laboratories, Italy) arrived 2-3 weeks prior to surgery, and were housed in a controlled environment (12 h light/dark cycle, 21±2°C, humidity 50±10%). Each animal was identified by an implantable subcutaneous microchip (PetID Microchip, E-vet, Haderslev, Denmark). Uninephrectomy was performed in 8-week-old *db/db* mice, as described in detail previously (Sembach et al., 2019); however, in this study, we removed the right kidney instead of the left kidney. Age-matched unoperated *db/m* mice served as healthy controls. The *db/db* UNx mice were randomized based on body weight and blood glucose levels, and received either a single injection of 1×10^10^ genome copies (GC) LacZAAV (UNx), which served as a negative control, or 2×10^10^ GC ReninAAV (UNx-Renin) at 12 weeks of age (Vector Biolabs, Malvern, PA, USA) (Harlan et al., 2018b). Mice had *ad libitum* access to tap water and chow (3.22 kcal/g, Altromin 1324, Brogaarden, Hoersholm, Denmark). Animals were terminated by cardiac puncture under isoflurane anesthesia 12 weeks after the injection of viral vector. After dissection, the kidney was cut sagitally and the two halves collected for histopathological evaluation and RNAseq.

### Blood pressure

At study week 4 and 10, systolic arterial blood pressure was measured using tail cuff plethysmography by a mouse tail cuff system (IITC Life Science, Woodland Hills, CA, USA). Animals (*n*=5-6) were randomly selected for blood pressure measurement and acclimated to the system for 4 consecutive days prior to the actual data acquisition on day 5.

### Blood and urine analyses

Blood glucose was measured throughout the study by collecting blood from the tail vein of nonfasted mice into heparinized glass capillary tubes and immediately suspending in glucose/lactate system solution buffer (EKF Diagnostics, Barleben, Germany). Blood glucose was measured using a BIOSEN c-Line glucose meter (EKF Diagnostics) according to the manufacturer's instructions. Spot urine samples were collected 6 and 12 weeks after the injection of viral vector directly from the vulva to determine the urine ACR. Urine creatinine was measured using the CREP2 kit (Roche Diagnostics, Mannheim, Germany) on a Cobas C-501 autoanalyzer. Urine albumin was measured using a Mouse Albumin ELISA Kit (Bethyl Laboratories, Montgomery, TX, USA).

### Histopathology

Kidney samples were fixated for 24 h in a 3.3% glyoxal solution (Sigma-Aldrich, Denmark) at 4°C. Samples were embedded in blocks of paraffin and 3 µm kidney sections were cut on a microtome. PAS staining was performed using standard procedures. Briefly, kidney sections were deparaffinized and oxidized in 0.5% periodic acid (Sigma-Aldrich, Denmark). Next, sections were incubated with Schiff's reagent (Sigma-Aldrich, Denmark) and counterstained with Mayer's Hematoxylin (Dako, Denmark). Sections were dehydrated and mounted with Pertex (Histolab, Sweden). PAS-stained slides were scanned in a Scanscope AT slide scanner (Leica, Denmark) under a 20× objective.

Total glomerular PAS-positive staining was determined by AI-assisted image analysis using the VIS software package (version 2020.01.3, Visiopharm, Denmark). Scanned slides were analysed using a two-step protocol. First, all glomeruli were detected using a trained U-NET network architecture. Next, PAS-positive surface area of the glomerular tuft was segmented using a simple threshold after PAS colour deconvolution. Total PAS mass was expressed as total mass (mg) of positive staining by multiplying the glomerular PAS-positive fractional area (percentage) by the terminal kidney weight.

### Laser capture microdissection

Frozen kidneys were sectioned (20 µm) on a cryostat (CM3050 S, Leica, Germany) and collected on PEN membrane glass slides (LCM0522, Thermo Fisher Scientific, Waltham, MA, USA) for LCM. Tissue-mounted slides were stored at −80°C until further processing.

Before LCM, sections were thawed for 5 min at 4°C and then dipped in ice-cold 70% ethanol to remove any remaining optimal cutting temperature compound (TissueTek OCT., Sakura Finetek, Denmark) from the cryosectioning. The sections were stained for 5 min in a 0.1% Cresyl Violet Acetate (Sigma-Aldrich, Søborg, Denmark) solution dissolved in 70% ethanol. After staining, sections were dehydrated once in 96% ethanol and twice in 100% ethanol for 30 s each at 4°C, and finally allowed to dry in a fume hood for 2 min at room temperature. LCM was then performed using Arcturus Veritas LCM equipment (Life Technologies, Carlsbad, CA, USA). The glomeruli were identified under a microscope and captured by a combination of infrared laser capturing and ultraviolet laser cutting. The glomeruli were captured using CapSure Macro LCM Caps (LCM0211, Thermo Fisher Scientific, Waltham, MA, USA). A total of 200 glomeruli were isolated per animal sample. The CapSure Macro LCM cap with captured tissue was inserted on to a 0.5 ml Eppendorf tube (022431005, Eppendorf, Germany) with 50 µl of extraction buffer (Arcturus PicoPure RNA Isolation Kit, KIT0204, Thermo Fisher Scientific, Waltham, MA, USA). The tube was inverted to make sure the tissue was covered by the extraction buffer. The tube with the LCM cap was incubated for 30 min at 42°C to ensure sufficient lysis. After lysis, the lysate was spun down, the LCM cap was removed and the tube was stored at −80°C until RNA purification.

### RNA purification of LCM samples

RNA extraction was performed using an Arcturus PicoPure RNA Isolation Kit, following the protocol recommended by manufacturer. Samples were treated with DNase for 10 min (AM1906, Thermo Fisher Scientific, Waltham, MA, USA) before adding stop solution. RNA was purified over two rounds using a total of 20 µl of RNase-free water. RNA quantity was determined using a NanoDrop (NanoDrop 2000/2000c, Thermo Fisher Scientific, Waltham, MA, USA), as recommended by the manufacturer. RNA quality was determined using an Agilent Bioanalyzer with an RNA 6000 Pico Kit (5067-1513, Agilent, Santa Clara, CA, USA), as recommended by the manufacturer. The RNA samples were then stored at −80°C until further processing.

### RNA purification of kidney cortex samples

RNA from snap-frozen kidney cortex samples (∼15 mg per animal) stored at −80°C was extracted using a NucleoSpin 8 RNA kit (Macherey-Nagel) and a vacuum manifold. The RNA quantity was measured using a NanoDrop, and RNA quality was determined using an Agilent Bioanalyzer with an RNA 6000 Nano Kit (5067-1511, Agilent, Santa Clara, CA, USA).

### Library preparation and sequencing of kidney cortex and LCM samples

cDNA library preparation was performed using an NEBNext Ultra II Directional RNA Library Prep Kit for Illumina (E7760L, NEG, Ipswich, MA, USA). The number of cycles used for cDNA amplification was 16 for kidney cortex samples and 18 for LCM samples. The sequencing of cDNA libraries was performed using an NS 500 high Output Kit v2 (75 cycles) (Illumina, San Diego, CA, USA) on an Illumina NextSeq 500 platform (Illumina, San Diego, CA, USA). The gene expression level is displayed as reads per kilobase million (RPKM), thus quantifying gene expression from mRNA sequencing data by normalizing for total read length and the number of sequencing reads.

Reads were mapped to the GRCm38 v96 Ensembl *Mus musculus* genome using Spliced Transcripts Alignment to a Reference (STAR) v.2.5.2a (Dobin et al., 2013). The R-package DESeq. 2 v.1.18.1 was used for differential expression analysis (Love et al., 2014). *P*-values were adjusted using the Benjamini–Hochberg method and genes with adjusted *P*<0.05 were considered statistically significantly regulated. The Reactome Pathway Database (Fabregat et al., 2018) was used for gene annotation for gene set analysis using the R package PIANO v.1.18.1, with the Stouffer method and Benjamini–Hochberg-adjusted *P*-values.

### Single nuclei isolation

Nuclei were isolated from kidney cortex samples from *db/m* (*n*=1) and UNx-Renin mice (*n*=2) using Nuclei EZ Lysis Buffer (NUC-101, Sigma-Aldrich) supplemented with Protector RNase Inhibitor (Sigma-Aldrich, 3335399001) (lysis buffer). Kidney samples were homogenized using a Dounce homogenizer in 1 ml of ice-cold lysis buffer. The homogenate was incubated on ice for 5 min with an additional 1 ml of lysis buffer and centrifuged at 500 ***g*** for 5 min at 4°C. The pellet was resuspended in 500 μl lysis buffer, and then incubated on ice for 5 min and centrifuged at 500 ***g*** for 5 min at 4°C. The pellet was resuspended in 1000 μl Nuclei Suspension Buffer [NSB, 1× PBS, 1% BSA (w/v), 0.27% Protector RNase Inhibitor] and centrifuged at 500 ***g*** for 5 min at 4°C. The pellet was resuspended in 250 μl NSB and filtered through a 40 µM cell strainer (PluriSelect, SKU 43-10040-50) and an additional 150 μl of NSB was added to the filter. The nuclei enriched in NSB was counted and stained with DRAQ5 (5 μM) (Thermo Fisher Scientific, 62254) for nuclei isolation using a BD FACSAria III sorter (70 μm nozzle, 70 psi). A total of 10,000 nuclei were sorted and loaded onto the 10x Chromium (10x Genomics). Library construction was performed using the Chromium Next GEM Single Cell 3′ GEM, Library & Gel Bead Kit v3.1 (10x Genomics) according to manufacturer's instructions. The number of cycles used for cDNA amplification was 13. cDNA libraries were sequenced on a NextSeq500 using a NextSeq 500/550 High Output Kit v2.5 (150 cycles) (Illumina).

### snRNAseq transcriptomic analysis

Reads were mapped to the GRCm38 v96 Ensembl *Mus musculus* genome using CellRanger (v. 1.1.0, 10x Genomics) software. Cell Ranger uses STAR software to align. For each sample, a count matrix was generated using both intronic and extronic reads. Data processing, including normalization, variance stabilization and dimensionality reduction, was performed using the Seurat R package (v. 3.1.1; Stuart et al., 2019). The quality of the data was evaluated using standard RNAseq quality control parameters. Additionally, nuclei with a mitochondrial RNA content of >0.25% were removed from the dataset as nuclei data should not contain any mitochondrial transcripts. The sctransform method was used for data normalization and variance stabilization (Hafemeister and Satija, 2019). To account for sample variance, samples were integrated using the ‘IntegrateData’ function in Seurat. PCA was computed for the integrated dataset and the top 20 PCs were used as input for UMAP dimensionality reduction, in which single nuclei can be projected onto a two-dimensional UMAP space for visualization purposes. The integrated dataset was used to select a biologically relevant number of clusters, which included determining differential expression between neighbouring clusters, creating a K-nearest neighbour graph and performing modularity optimization (Louvain algorithm).

Differential expression between clusters was calculated using a likelihood-ratio test for single-cell gene expression implemented in Seurat at a family-wise error rate of 5%. Cell identity of the nuclei was determined by examining the most enriched genes in each cluster (marker genes) and comparing these with canonical marker genes. To associate bulk gene regulations with a specific cell type, we defined cell type-specific genes if the following criteria were fulfilled: (1) the gene should have the highest expression level in that cell type, and (2) the gene should have at least twofold increased expression compared to the cell type with the second highest expression level.

### Statistical analysis

Results are presented as mean±s.e.m. unless otherwise specified. Apart from RNAseq data sets, all data were analysed using GraphPad Prism software (version 9.0.0) using either one-way ANOVA with Tukey's post-hoc test (body weight, kidney weight and glomerulosclerosis) or two-way ANOVA with Bonferroni's post-hoc test (blood glucose and urine ACR). Urine ACR values were log10-transformed before group comparisons. *P*<0.05 was considered statistically significant.

## Supplementary Material

Supplementary information
