# Supplementary material for: Integrative transcriptomic profiling of a mouse model of hypertension-accelerated diabetic kidney disease
Source: Dis Model Mech. 2021 Oct 25;14(10):dmm049086. doi: 10.1242/dmm.049086 (PMC8560499; doi:10.1242/dmm.049086)
Supplement: Supplementary information [file dmm-14-049086-s1.pdf]

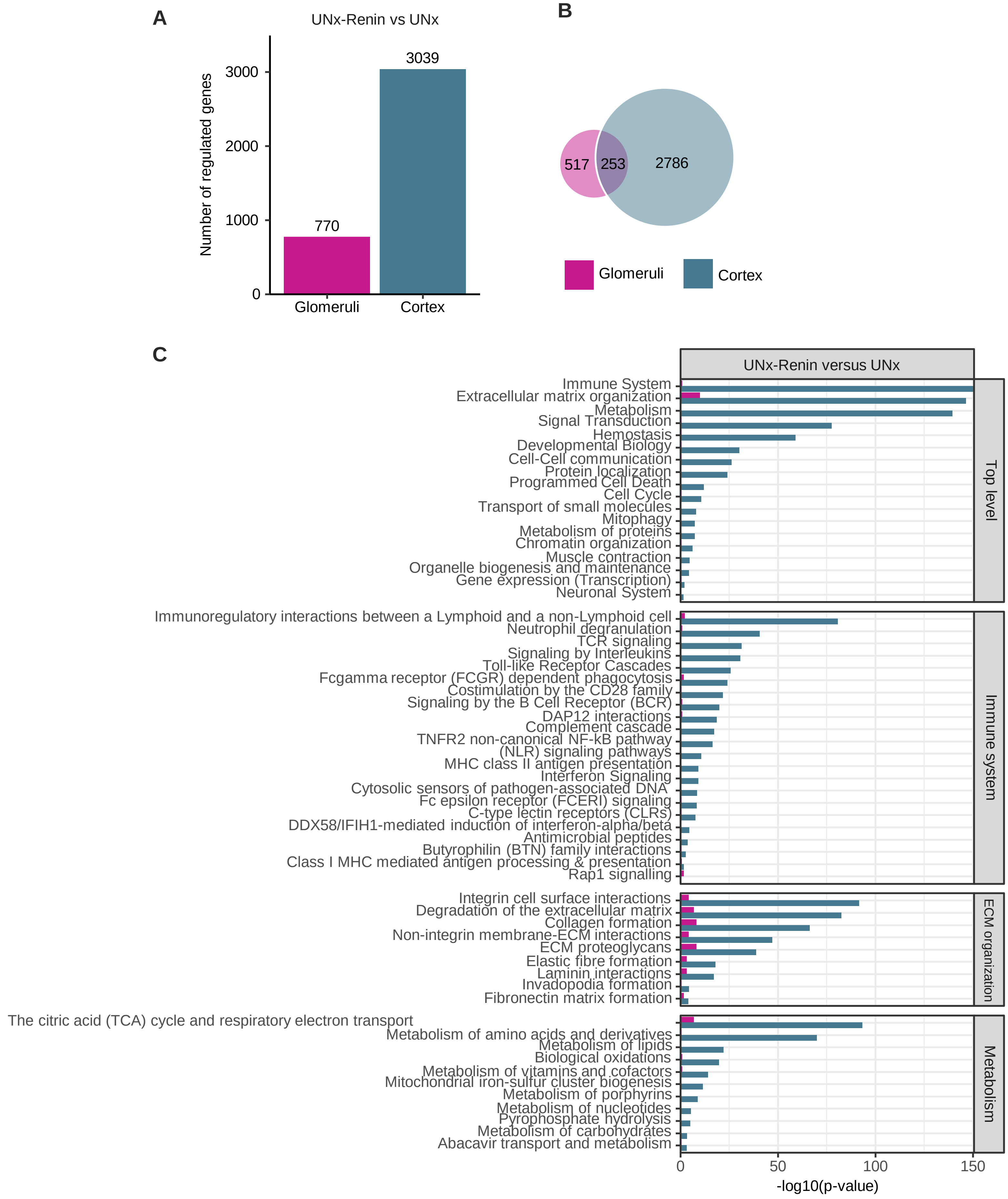

Fig. S1. Differentially expressed genes and gene enrichment analysis for UNx-Renin mice compared to UNx mice in both glomeruli and kidney cortex samples. (A) Total number of DEGs in glomeruli and kidney cortex in UNx-Renin mice compared to UNx mice. (B) Venn diagram depicting shared and separate DEGs in glomeruli and kidney cortex. (C) Reactome pathway gene enrichment analysis in glomeruli and kidney cortex. Degree of perturbation is presented as the −log10(p-value) after correction for gene-wise multiple testing (n=5-13).

Disease Models & Mechanisms • Supplementary information

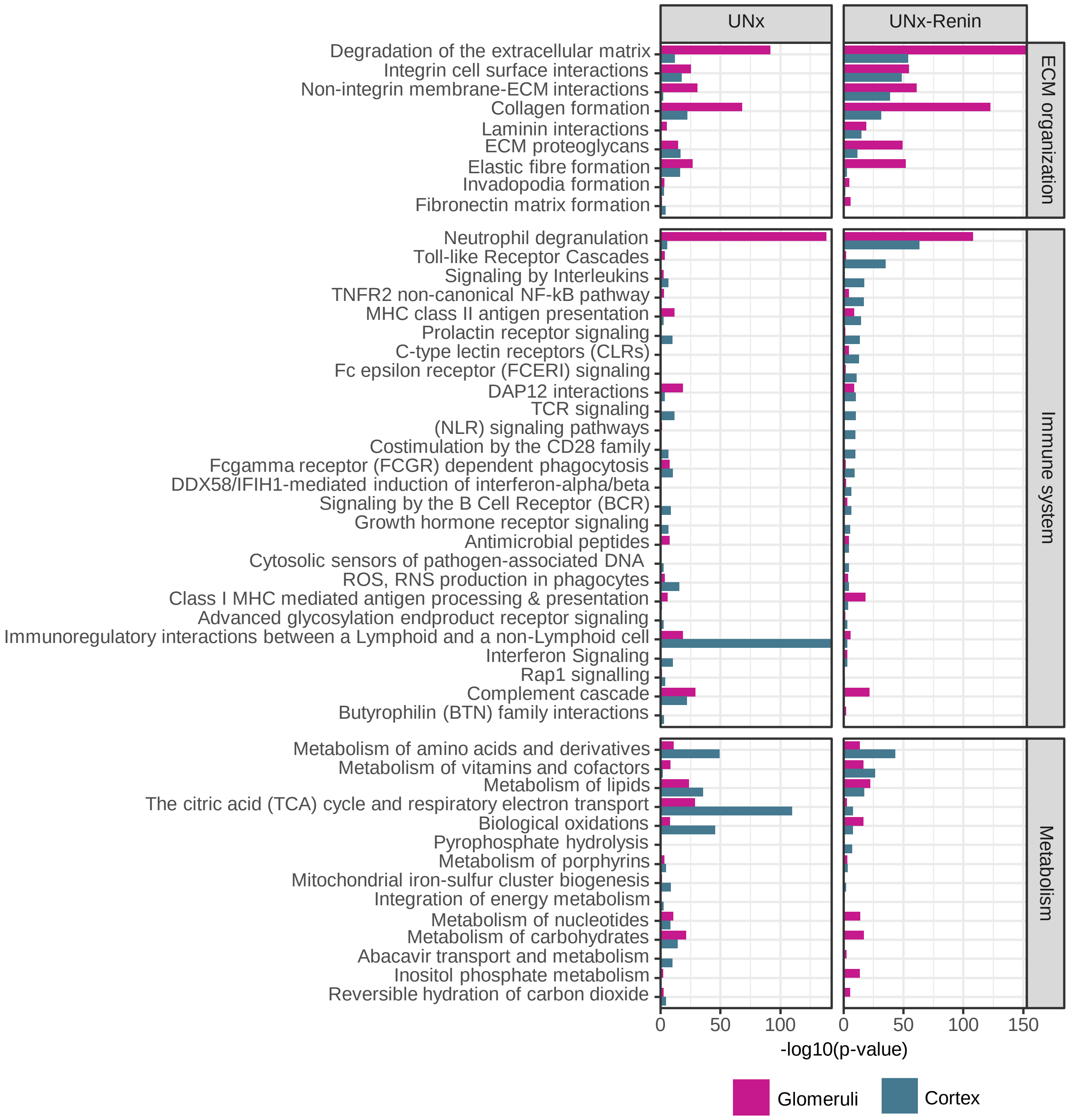

Fig. S2. Gene enrichment analysis using Reactome of sub-pathways for UNx or UNx-Renin mice compared to db/m controls. Degree of perturbation is presented as the −log10(p-value) after correction for gene-wise multiple testing (n=5-13).

Disease Models & Mechanisms • Supplementary information

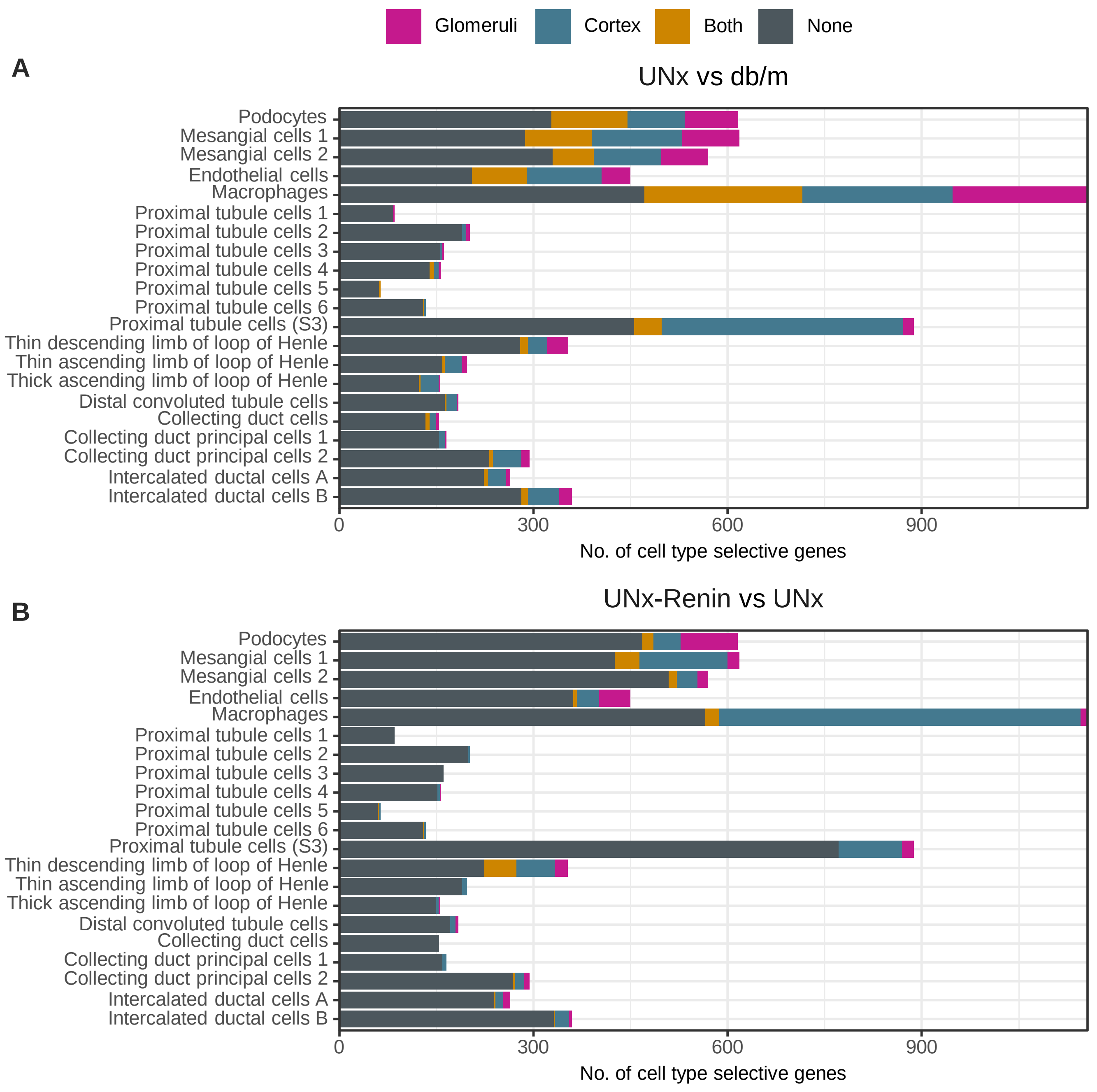

Fig. S3. DEGs mapped to specific cell types. (A) Number of cell type specific DEGs between UNx mice and db/m controls, or (B) between UNx-Renin and UNx mice, in glomeruli, cortex, both or none of the two tissue areas. Genes were defined as specific to the cell population with the highest average expression level, if the expression level was increased by 2-fold as compared to the cell population with the second highest expression level.

Disease Models & Mechanisms • Supplementary information

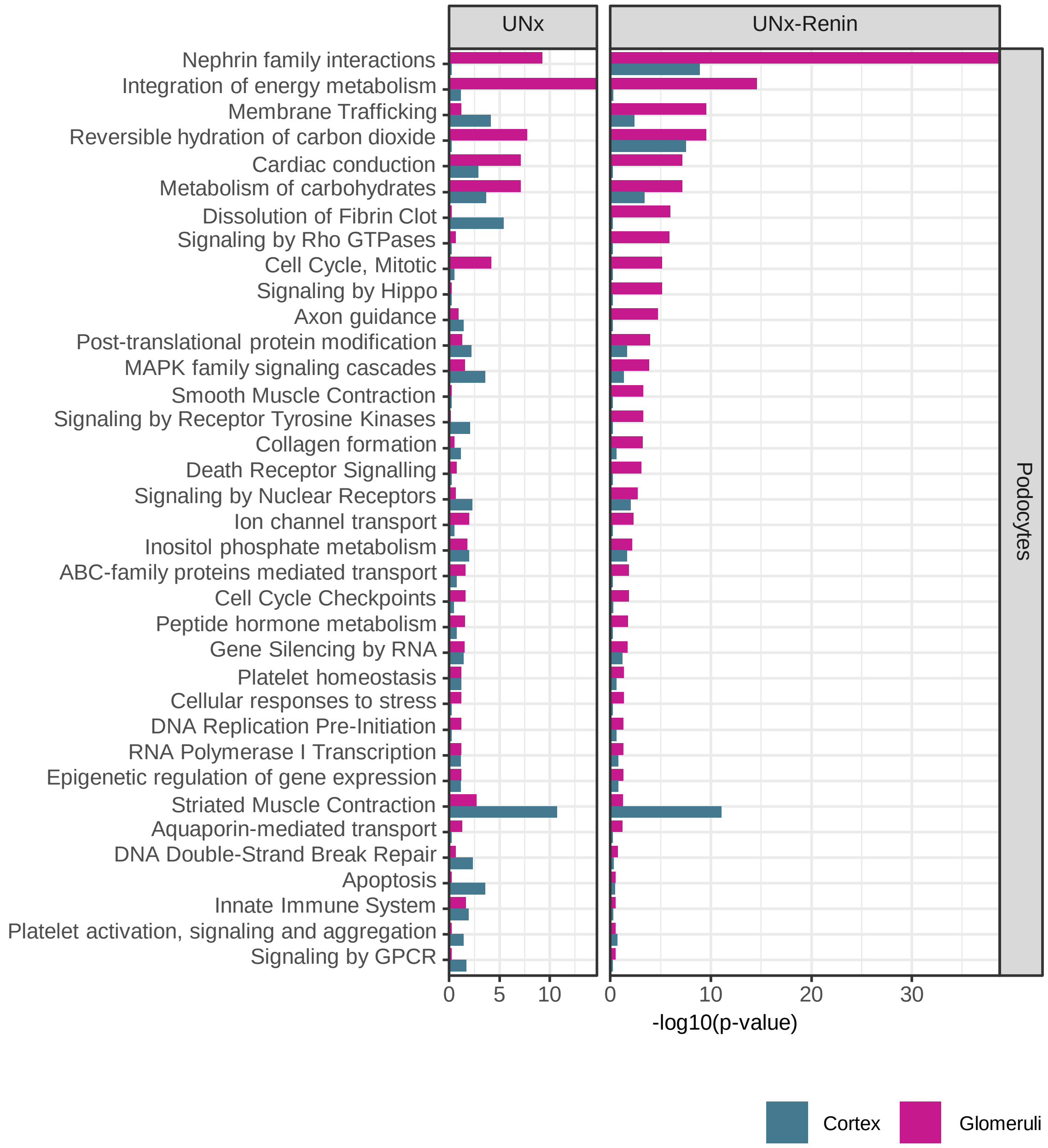

Fig. S4. Podocyte- specific DEGs found in kidney cortex and glomeruli of UNx and UNx-Renin mice compared to db/m controls. Degree of perturbation is presented as the −log10(p-value) after correction for gene-wise multiple testing (n=5-13).

Disease Models & Mechanisms • Supplementary information

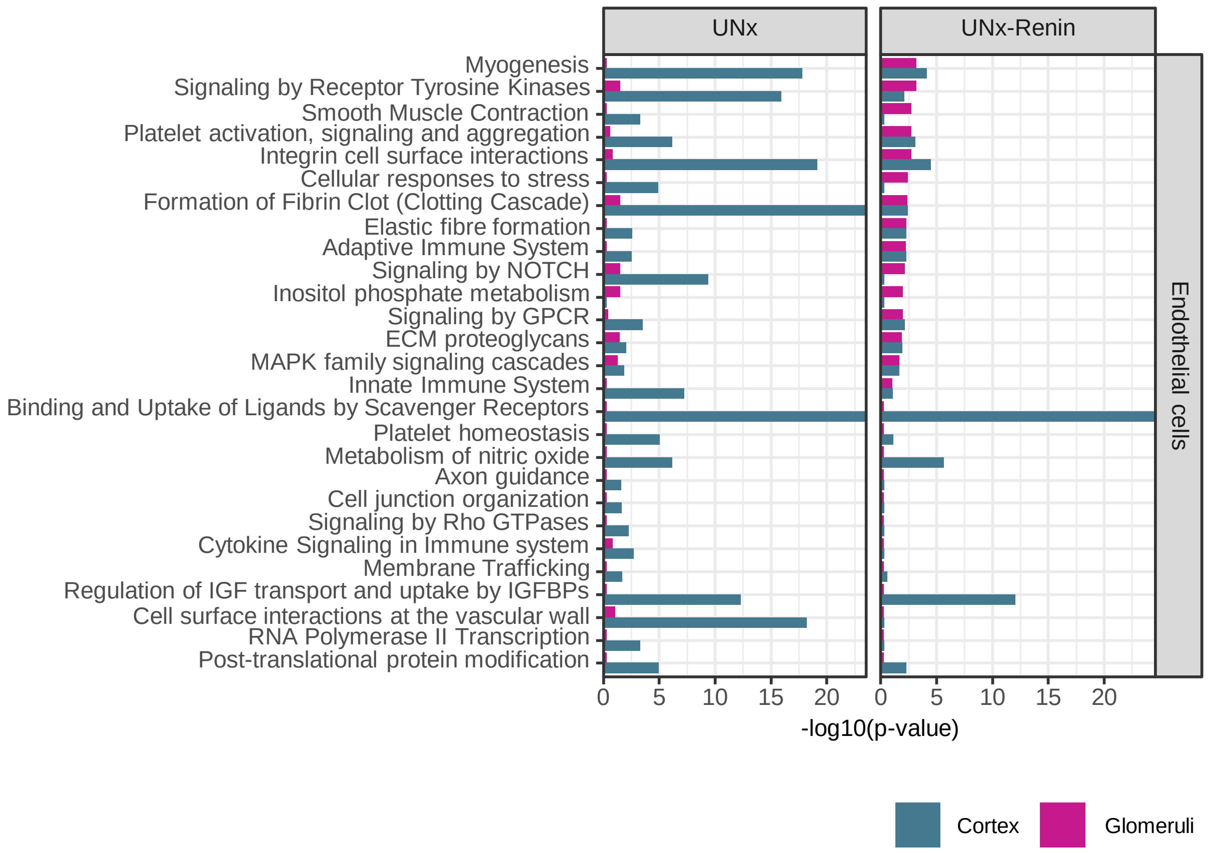

Fig. S5. Endothelial cell- specific DEGs found in kidney cortex and glomeruli of UNx and UNx-Renin mice compared to db/m controls. Degree of perturbation is presented as the −log10(p-value) after correction for gene-wise multiple testing (n=5-13).

Disease Models & Mechanisms • Supplementary information

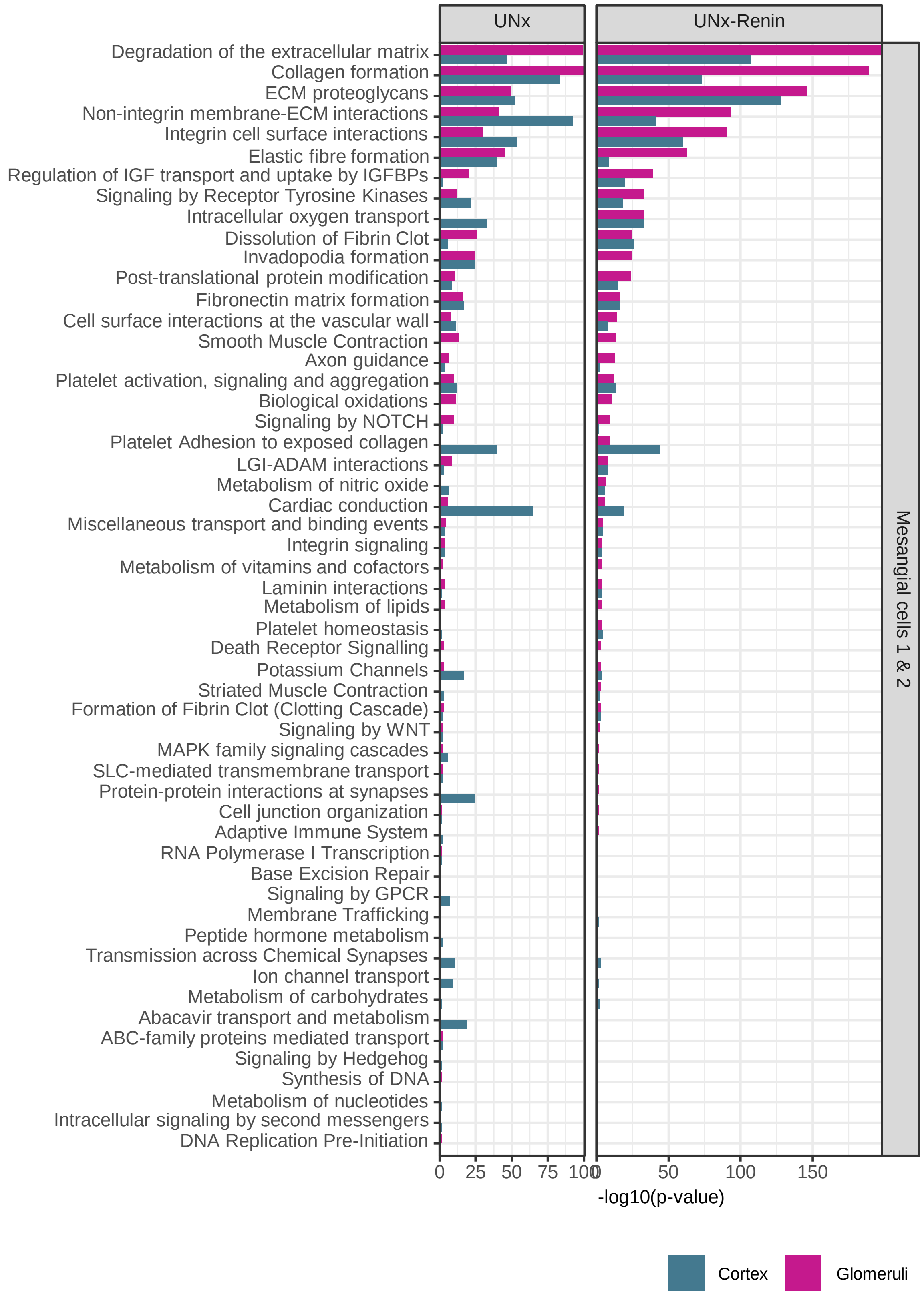

Fig. S6. Mesangial cell- specific DEGs found in kidney cortex and glomeruli of UNx and UNx-Renin mice compared to db/m controls. Degree of perturbation is presented as the −log10(p-value) after correction for gene-wise multiple testing (n=5-13).

Table S1. Selected differential expressed genes (DEGs) in kidney cortex

Summary of selected DEGs in kidney cortex and comparison to relevant transcriptomic studies of human DKD (Woroniecka *et al.*, 2011; Ju *et al.*, 2013; Levin *et al.*, 2020; Fan *et al.*, 2019; Verzola *et al.*, 2014; Sircar *et al.*, 2018) including use of the Nephroseq database. Fold change, FC; NA, not applicable; NS, non-significant.

Gene Name	Gene Description	UNx vs db/m	UNx-Renin vs db/m	UNx-Renin vs UNx	Human DKD Transcriptomics
		Log_2 FC (p-value)	Log_2 FC (p-value)	Log_2 FC (p-value)	
Adamts4	a disintegrin-like and metallopeptidase (reprolysin type) with thrombospondin type 1 motif, 4	0.5 (0.13)	1.3 (0.001)	0.8 (0.07)	NA
C3	complement component 3	0.0 (0.87)	1.8 (5.54E-11)	1.8 (7.08E-11)	Upregulated in tubulointerstitium in Woroniecka *et al.*, 2011 and Ju *et al.*, 2013.
C6	complement component 6	1.3 (0.0006)	2.7 (3.09E-10)	1.4 (0.002)	Upregulated in tubulointerstitium in Woroniecka *et al.*, 2011 and Ju *et al.*, 2013.
C7	complement component 7	0.9 (0.0004)	1.9 (1.98E-08)	1.0 (0.01)	Upregulated in tubulointerstitium in Woroniecka *et al.*, 2011; Ju *et al.*, 2013 and Sircar *et al.*, 2018.
Col1a1	collagen, type I, alpha 1	-0.9 (0.01)	1.1 (0.03)	2.0 (2.49E-05)	Upregulated in tubulointerstitium in Woroniecka *et al.*, 2011; Ju *et al.*, 2013 and Levin *et al.*, 2020.
Col3a1	collagen, type III, alpha 1	-1.4 (2.26E-05)	0.6 (0.25)	2.0 (1.38E-05)	Upregulated in tubulointerstitium in Woroniecka *et al.*, 2011 and Ju *et al.*, 2013.
Col5a1	collagen, type V, alpha 1	-0.7 (2.47E-08)	0.2 (0.25)	0.9 (6.83E-08)	Upregulated in tubulointerstitium in Woroniecka *et al.*, 2011 and Ju *et al.*, 2013.
Col5a3	collagen, type V, alpha 3	0.2 (0.47)	1.1 (7.61E-06)	0.9 (0.0004)	NS regulated in Ju *et al.*, 2013, while downregulated in Woroniecka *et al.*, 2011.

Disease Models & Mechanisms • Supplementary information

Col6a1	collagen, type VI, alpha 1	-0.2 (0.09)	0.6 (0.0001)	0.9 (2.36E-07)	Downregulated in tubulointerstitium in Woroniecka *et al.*, 2011 and Ju *et al.*, 2013.
Col6a2	collagen, type VI, alpha 2	-0.2 (0.24)	0.9 (6.16E-06)	1.1 (4.98E-08)	Upregulated in tubulointerstitium in Woroniecka *et al.*, 2011 and NS regulated in Ju *et al.*, 2013.
Cxcl1	chemokine (C-X-C motif) ligand 1	1.2 (0.08)	3.8 (3.23E-06)	2.6 (0.003)	Upregulated in whole kidney in Fan *et al.*, 2019 and in tubulointerstitium in Woroniecka *et al.*, 2011 and Ju *et al.*, 2013.
Cxcl2	chemokine (C-X-C motif) ligand 2	1.0 (0.21)	5.1 (2.28E-13)	4.2 (5.50E-10)	Downregulated in whole kidney in Fan *et al.*, 2019 and NS regulated in tubulointerstitium in Woroniecka *et al.*, 2011 and Ju *et al.*, 2013.
Cyp2d9	cytochrome P450, family 2, subfamily d, polypeptide 9	2.8 (3.72E-50)	2.4 (1.60E-20)	-0.4 (0.20)	NA
Fn1	fibronectin 1	-0.5 (0.008)	0.7 (0.008)	1.3 (2.25E-06)	Upregulated in tubulointerstitium in Woroniecka *et al.*, 2011 and Ju *et al.*, 2013.
Lox	lysyl oxidase	0.1 (0.81)	1.4 (8.50E-07)	1.3 (8.62E-06)	NS regulated in tubulointerstitium in Woroniecka *et al.*, 2011, while downregulated in Ju *et al.*, 2013.
Mapk12	mitogen-activated protein kinase 12	-0.5 (7.55E-08)	-0.7 (1.64E-08)	-0.2 (0.18)	NS regulated in tubulointerstitium in Ju *et al.*, 2013.
Mmp12	matrix metallopeptidase 12	0.8 (0.02)	3.2 (3.22E-23)	2.4 (2.78E-14)	NS regulated in tubulointerstitium in Woroniecka *et al.*, 2011 and Ju *et al.*, 2013.
Mmp14	matrix metallopeptidase 14 (membrane-inserted)	-0.3 (0.06)	0.7 (0.001)	1.1 (2.05E-06)	NS regulated in tubulointerstitium in Ju *et al.*, 2013, while downregulated in Woroniecka *et al.*, 2011.
Mmp3	matrix metallopeptidase 3	-0.1 (0.86)	1.1 (0.16)	1.3 (0.13)	NS regulated in tubulointerstitium in Woroniecka *et al.*, 2011 and Ju *et al.*, 2013.

Disease Models & Mechanisms • Supplementary information

Mmp7	matrix metallopeptidase 7	-2.1 (0.16)	2.7 (0.15)	4.8 (0.01)	Upregulated in whole kidney in Fan *et al.*, 2019 and in tubulointerstitium in Woroniecka *et al.*, 2011 and Ju *et al.*, 2013.
Mmp8	matrix metallopeptidase 8	0.9 (0.42)	2.2 (0.08)	1.2 (0.36)	NS regulated in tubulointerstitium in Ju *et al.*, 2013, while downregulated in Woroniecka *et al.*, 2011.
Nphs1	nephrosis 1, nephrin	-0.1 (0.59)	-0.5 (0.001)	-0.4 (0.01)	Downregulated in tubulointerstitium in Woroniecka *et al.*, 2011 and Ju *et al.*, 2013.
Nphs2	nephrosis 2, podocin	0.1 (0.58)	-0.1 (0.81)	-0.1 (0.50)	NS regulated in tubulointerstitium in Woroniecka *et al.*, 2011, while downregulated in Ju *et al.*, 2013.
Rbp2	retinol binding protein 2, cellular	3.4 (0.0003)	2.7 (0.04)	-0.7 (0.63)	NA
Serpine1	serine (or cysteine) peptidase inhibitor, clade E, member 1	0.0 (0.89)	1.1 (0.0001)	1.0 (0.0004)	Downregulated in tubulointerstitium in Woroniecka *et al.*, 2011 and Ju *et al.*, 2013.
Timp1	tissue inhibitor of metalloproteinase 1	-0.6 (0.51)	2.1 (0.04)	2.6 (0.008)	Upregulated in tubulointerstitium in Woroniecka *et al.*, 2011 and Ju *et al.*, 2013.
Tlr4	toll-like receptor 4	-0.1 (0.52)	0.6 (0.03)	0.7 (0.006)	Upregulated in tubulointerstitium in Verzola *et al.*, 2014, and Ju *et al.*, 2013.
Ugt1a10	UDP glycosyltransferase 1 family, polypeptide A10	1.8 (3.58E-30)	1.8 (2.08E-18)	0.1 (0.88)	NA
Vcam1	vascular cell adhesion molecule 1	-0.4 (0.02)	2.4 (3.57E-29)	2.8 (2.53E-40)	Upregulated in tubulointerstitium in Woroniecka *et al.*, 2011 and Ju *et al.*, 2013.

Disease Models & Mechanisms • Supplementary information

Table S2. Selected differential expressed genes (DEGs) in glomeruli

Summary of selected DEGs in glomeruli and comparison to relevant transcriptomic studies of human DKD (Woroniecka *et al.*, 2011; Ju *et al.*, 2013; Levin *et al.*, 2020; Verzola *et al*., 2014) including use of the Nephroseq database. Fold change, FC; NA, not applicable; NS, non-significant.

Gene Name	Gene Description	UNx vs db/m	UNx-Renin vs db/m	UNx-Renin vs UNx	Comments
		Log_2 FC (p-value)	Log_2 FC (p-value)	Log_2 FC (p-value)	
Adamts4	a disintegrin-like and metallopeptidase (reprolysin type) with thrombospondin type 1 motif, 4	3.8 (1.39E-11)	3.4 (1.89E-09)	-0.4 (0.74)	NA
C3	complement component 3	-0.1 (0.94)	2.2 (1.48E-05)	2.3 (5.39E-05)	Upregulated in glomeruli in Woroniecka *et al.*, 2011; Ju *et al.*, 2013 and Levin *et al.*, 2020.
C6	complement component 6	5.2 (1.22E-08)	3.0 (0.003)	-2.2 (0.03)	NS regulated in glomeruli in Woroniecka *et al.*, 2011, while downregulated in Ju et al., 2013.
C7	complement component 7	1.4 (0.30)	2.1 (0.08)	0.6 (0.81)	Upregulated in glomeruli in Woroniecka *et al.*, 2011, while NS regulated in Ju et al., 2013.
Col1a1	collagen, type I, alpha 1	1.1 (0.37)	2.7 (0.003)	1.7 (0.25)	Upregulated in glomeruli in Woroniecka *et al.*, 2011; Ju *et al.*, 2013 and Levin *et al.*, 2020.
Col3a1	collagen, type III, alpha 1	0.7 (0.12)	2.1 (8.98E-09)	1.4 (0.002)	Upregulated in glomeruli in Ju *et al.*, 2013, while NS regulated in Woroniecka *et al.*, 2011.
Col5a1	collagen, type V, alpha 1	1.0 (8.24E-16)	1.4 (1.54E-31)	0.4 (0.01)	Upregulated in glomeruli in Woroniecka *et al.*, 2011 and Ju *et al.*, 2013.
Col5a3	collagen, type V, alpha 3	3.3 (2.74E-10)	5.2 (1.03E-25)	1.9 (0.0002)	NS regulated in glomeruli in Woroniecka *et al.*, 2011 and Ju *et al.*, 2013.
Col6a1	collagen, type VI, alpha 1	-0.3 (0.52)	2.0 (4.74E-08)	2.3 (8.54E-10)	Upregulated in glomeruli in Woroniecka *et al.*, 2011, while NS regulated in Ju *et al.*, 2013.

Disease Models & Mechanisms • Supplementary information

Col6a2	collagen, type VI, alpha 2	0.0 (0.97)	1.7 (8.35E-06)	1.7 (8.50E-05)	Upregulated in glomeruli in Woroniecka *et al.*, 2011 and Ju *et al.*, 2013.
Cxcl1	chemokine (C-X-C motif) ligand 1	1.4 (0.01)	3.1 (5.48E-10)	1.6 (0.01)	Upregulated in glomeruli in Ju *et al.*, 2013, while NS regulated in Woroniecka *et al.*, 2011.
Cxcl2	chemokine (C-X-C motif) ligand 2	1.3 (0.20)	3.4 (2.19E-05)	2.1 (0.04)	NS regulated in glomeruli in Woroniecka *et al.*, 2011 and Ju *et al.*, 2013.
Cyp2d9	cytochrome P450, family 2, subfamily d, polypeptide 9	1.9 (0.22)	1.3 (0.43)	-0.6 (0.86)	NA
Fn1	fibronectin 1	2.7 (1.58E-07)	4.2 (8.82E-19)	1.6 (0.01)	Upregulated in glomeruli in Woroniecka *et al.*, 2011 and Ju *et al.*, 2013.
Lox	lysyl oxidase	1.3 (0.0001)	3.2 (1.89E-29)	2.0 (2.39E-11)	Downregulated in glomeruli in Woroniecka *et al.*, 2011 and Ju *et al.*, 2013.
Mapk12	mitogen-activated protein kinase 12	-0.1 (0.51)	-0.5 (0.0002)	-0.4 (0.03)	NS regulated in glomeruli in Ju *et al.*, 2013.
Mmp12	matrix metallopeptidase 12	5.7 (3.78E-36)	6.3 (8.38E-46)	0.7 (0.08)	NS regulated in glomeruli in Woroniecka *et al.*, 2011 and Ju *et al.*, 2013.
Mmp14	matrix metallopeptidase 14 (membrane-inserted)	0.7 (1.74E-10)	0.8 (6.79E-15)	0.1 (0.59)	NS regulated in glomeruli in Woroniecka *et al.*, 2011, while downregulated in Ju et al., 2013.
Mmp3	matrix metallopeptidase 3	2.1 (1.96E-05)	2.2 (4.96E-06)	0.1 (0.96)	in glomeruli in Woroniecka *et al.*, 2011, while NS regulated in Ju *et al.*, 2013.
Mmp7	matrix metallopeptidase 7	3.6 (0.27)	6.4 (0.02)	2.8 (0.58)	Upregulated in glomeruli in Woroniecka *et al.*, 2011 and Ju *et al.*, 2013.
Mmp8	matrix metallopeptidase 8	3.7 (2.48E-06)	3.0 (0.0002)	-0.7 (0.69)	NS regulated in glomeruli in Woroniecka *et al.*, 2011.
Nphs1	nephrosis 1, nephrin	-0.3 (0.02)	-0.8 (3.99E-16)	-0.6 (1.86E-06)	Downregulated in glomeruli in Woroniecka *et al.*, 2011 and Ju *et al.*, 2013.
Nphs2	nephrosis 2, podocin	0.1 (0.40)	-0.4 (0.002)	-0.5 (7.09E-05)	Downregulated in glomeruli in Woroniecka *et al.*, 2011, while NS regulated in Ju *et al.*, 2013.
Rbp2	retinol binding protein 2, cellular	3.0 (0.24)	0.9 (0.73)	-2.1 (0.42)	NA
Serpine1	serine (or cysteine) peptidase inhibitor, clade E, member 1	0.6 (0.02)	2.1 (1.58E-26)	1.5 (2.38E-13)	NS regulated in glomeruli in Woroniecka *et al.*, 2011.
Timp1	tissue inhibitor of metalloproteinase 1	2.0 (0.0007)	3.0 (3.01E-08)	1.0 (0.26)	NS regulated in glomeruli in Woroniecka *et al.*, 2011, while upregulated in Ju *et al.*, 2013.
Tlr4	toll-like receptor 4	0.6 (0.005)	0.7 (0.0002)	0.1 (0.75)	NS regulated in Ju *et al.,* 2013, but upregulated in Verzola *et al.*, 2014.
Ugt1a10	UDP glycosyltransferase 1 family, polypeptide A10	1.4 (0.68)	2.8 (0.41)	1.3 (0.69)	NA
Vcam1	vascular cell adhesion molecule 1	-0.4 (0.08)	0.4 (0.04)	0.8 (0.0001)	Upregulated in glomeruli in Woroniecka *et al.*, 2011 and Ju *et al.*, 2013.
